# Imaging and clinical features of giant multilocular prostatic cystadenoma: A case report

**DOI:** 10.1097/MD.0000000000022666

**Published:** 2020-10-09

**Authors:** Jingya Chen, Wei Zhang, Hu Chen, Yajing Wang, Zhengjun Li, Huiming Wu, Jian Zhang, Zhongqiu Wang

**Affiliations:** a Department of Radiology, Affiliated Hospital of Nanjing University of Chinese Medicine, Nanjing; b Department of Radiology, Affiliated Hospital of Integrated Traditional Chinese and Western medicine, Nanjing University of Chinese Medicine; c Department of Radiology, Liyang Traditional Chinese Medicine Hospital, Jiangsu China.

**Keywords:** computed tomography, giant multilocular prostatic cystadenoma, magnetic resonance imaging, prostate

## Abstract

**Rationale:**

Giant multilocular prostatic cystadenoma (GMPC) is a rare type of prostatic epithelial neoplasm. Thus, the imaging features of this condition are not well known. We report the imaging and clinical manifestations of a case of GMPC.

**Patient concerns:**

The case reported here relates to a 71-year-old man who complained of urination frequency and excessive urination at night. He underwent computed tomography (CT) and magnetic resonance imaging (MRI) examination before surgery, both tests revealed a mass body in the prostate.

**Diagnosis:**

Ultrasound-guided fine needle aspiration was performed and a diagnosis of GMPC was made by histological examination.

**Interventions:**

The patient received radical pelvic tumor resection successfully.

**Outcomes:**

Two months after surgery, the follow-up CT and magnetic MRI re-examination found no signs of recurrence.

**Lessons:**

GMPC is a rare prostatic neoplasm with atypical clinical symptoms. MRI provides valuable information about GMPC. In case of a giant multilocular prostatic mass with well-defined boundary and abundant vascularity, benign feature on diffusion-weighted imaging, GMPC should be considered.

## 1 Introduction

Giant multilocular prostatic cystadenoma (GMPC) is a rare benign tumor arising from the prostate gland. The tumor commonly presents as a locular cyst-solid mass in the pelvic. It was first reported by Watanabe et al^[1]^ in 1990. Since then, less than 40 cases have been reported. Imaging examination especially by magnetic resonance imaging (MRI) can reveal not only the components of the tumor but also the condition of adjacent organs. This provides valuable information for accurate diagnosis and differential diagnosis of GMPC. We herein report the imaging, clinical, and histologic findings of a case of GMPC and provide a review of literature on this condition.

## 2 Case report

### 2.1 Consent

This study was approved by the investigational review board of affiliated hospital of Chinese medicine. Informed written consent was obtained from the patient for publication of this case report and accompanying images.

### 2.2 Clinical and laboratory data

A 71-year-old man presented with a 3-month history of urination frequency and excessive urination at night. He reported no history of hematuria, fever, or pain. Digital rectal examination showed an enlarged prostate with disappeared central sulcus, no obvious nodules were seen, the boundary of the prostate was unclear. Laboratory tests revealed many abnormal findings. Serum total prostate-specific antigen (TPSA) was 47 ng/mL (normal value 0–4 ng/mL); free prostate-specific antigen (FPSA) was 3.96 ng/mL (0–2.5 ng/mL); creatinine was 120. 2 μmol/L (44–110 μmol/L). A urine test showed positive urine protein.

### 2.3 Computed tomography and magnetic resonance imaging

**Figure 1:**
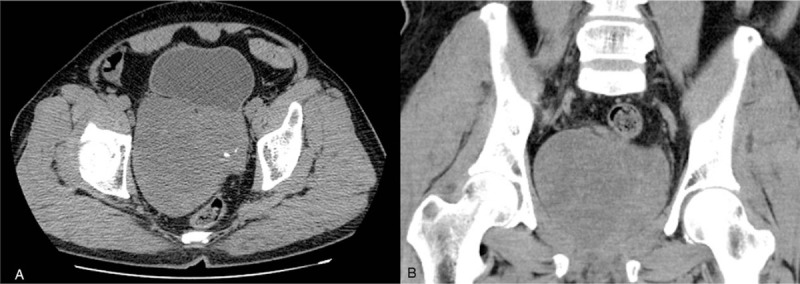
CT imaging of the GMPC. Axial (A) and coronal conventional CT scan reveals a multilobulated soft tissue mass in the pelvic with low-density area in the tumor. GMPC = giant multilocular prostatic cystadenoma, CT = computed tomography.

A large multilocular cystic-solid mass was observed in the pelvic cavity with a partially ill-defined border in nonenhanced computed tomography (CT) image (Fig. 1). The tumor was approximately 12 cm × 11 cm in size. The mass was heterogeneous in density. Cystic and soft tissue components were separated by multiple septations, accompanied with punctate calcifications in the tumor parenchyma. The bladder was displaced by the tumor, but the boundary between them was clear. The shape of the prostate and bilateral seminal vesicle glands was obscure, indicating that the tumor originated from the prostate.

**Figure 2:**
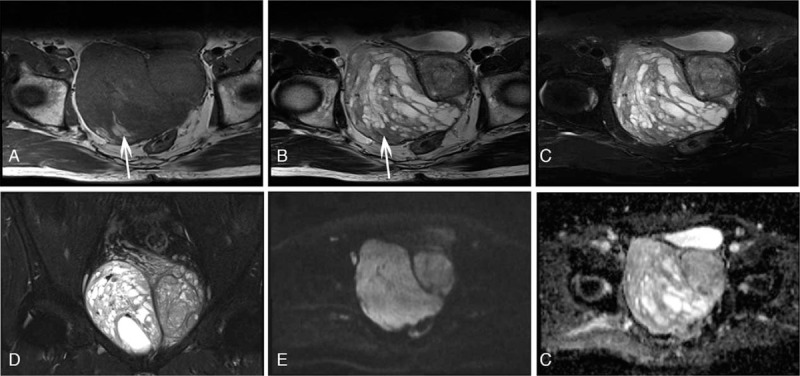
MRI features of the tumor. The tumor appeared as isointense on T1WI (A). The solid part of the tumor showed slightly higher SI on T2WI (B), and most of the cysts of the tumor showed hyperintense SI on T2WI. Some cysts that showed high SI on T1WI were hypointense on T2WI (arrow). C and D, T2WI fat-suppressed imaging showed the tumor was composed as a solid-cystic mass in the pelvic. The solid part of the tumor was slightly higher SI on DWI (b = 800) and ADC maps (E, F). ADC = apparent diffusion coefficient, DWI = diffusion-weighted imaging, MRI = magnetic resonance imaging, SI = signal intensity, T1WI = T1-weighted imaging, T2WI = T2-weighted imaging.

**Figure 3:**
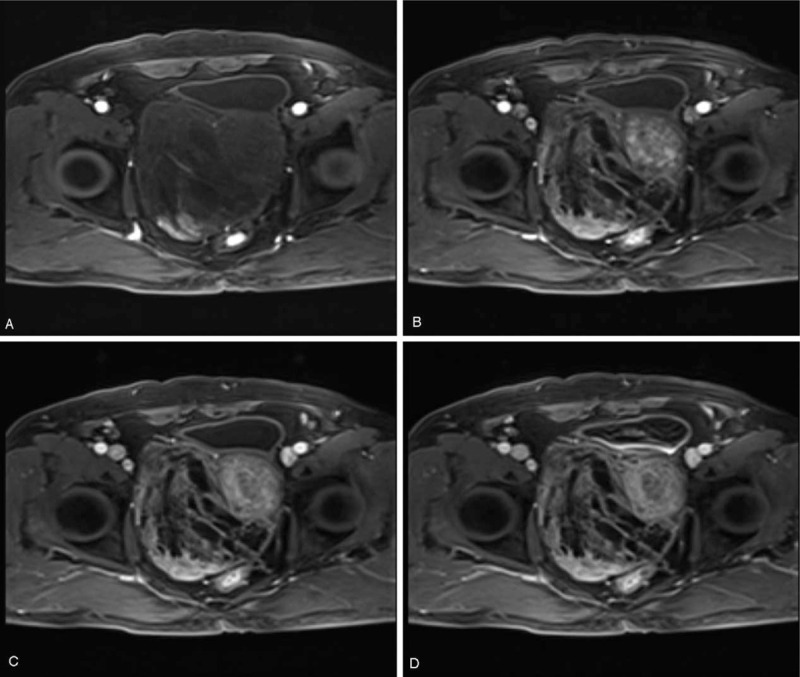
DCE-MRI manifestation of GMPC. On DCE-MRI, the solid part of the tumor showed obvious enhancement, and the cysts showed nonenhancement during arterial (A) phase to delayed phase (D). MRI = magnetic resonance imaging, DCE-MRI = dynamic contrast-enhanced MRI, GMPC = giant multilocular prostatic cystadenoma.

The tumor manifested heterogeneous signal intensity (SI) on T1-weighted imaging (T1WI) and T2-weighted imaging (T2WI). Most areas of the tumor were isointense with surrounding muscles on T1WI with patchy high SI areas (Fig. 2A). A multiple cystic mass was seen in the tumor on T2WI which was separated by multiple thick septa (Fig. 2, B–D). The high SI areas on T1WI showed low or high SI on T2WI according to mucinous or blood components. Solid part of the tumor showed slightly higher SI on diffusion-weighted imaging (DWI) and apparent diffusion coefficient (ADC) maps (Fig. 2, E and F). Contrast-enhanced MRI showed enhanced solid part and septa, but no enhancement of the cyst cavity (Fig. 3). The structure of seminal vesicles was not clear, the prostate was displaced to the left and bladder filling was impaired.

### 2.4 Surgery and histopathologic evaluation

Fine needle biopsy was performed under ultrasound guidance, which found no obvious evidence of malignancy. The patient underwent radical pelvic tumor resection. A cystic-solid mass was identified in the right pelvic floor, and prostate and seminal vesicle adhered to each other. Examination of the specimen revealed a 12 cm × 9 cm × 6.5 cm gray-red mass. The surface was covered with an incomplete capsule. The cut surface appeared gray-white, and various sized cysts were filled with brown or milky liquid. Some areas were honeycomb-shaped. Histologically, adenoid and cystic structures in a fibrous stroma were seen in the tumor. Immunohistochemical examination showed that epithelial cells of the neoplasm were positive for prostate-specific antigen (PSA), NKX3.1 and prostate acid phosphatase (PSAP); basal cells were positive for P63 and high molecular weight cytokeratin; stromal cells were positive for CD34, smooth muscle antibody, Desmin, estrogen receptor, and prolactin receptor. Collectively, these led to the diagnosis of multilocular prostate cystadenoma.

TPSA and FPSA were normal (6.31 ng/mL and 1.30 ng/mL, respectively) after 2 months of surgery. CT and MRI re-examination found no signs of recurrence.

## 3 Discussion

GMPC is a rare benign neoplasm of prostatic origin. It is characterized by significantly enlarged prostatic cystadenoma, most of them are larger than 10 cm in size (about 68% in the literature).^[2–15]^ In this condition, the tumor always originates from the prostate and extends to the space between the rectum and bladder, sometimes it may attach to the prostate with pedicle or even be completely separated from it. The tumor boundary is usually clear without invasion to the adjacent tissues and organs. This disease may appear at any age, but it is more commonly seen in 35 to 65 years old. It often manifests as lower abdominal masses or progressive dysuria, sometimes present with frequent urination.^[7]^ Radical excision is recommended for this condition. Patients will have an excellent prognosis and recurrence is rare if the tumor is totally removed.

Most of the clinical symptoms arise from localized effects of the mass, including palpable abdomen mass, oliguria, intermittent urination, and acute urinary retention.^[11]^ Occasionally, constipation caused by mechanical compression of the rectum can be seen.^[8]^ These manifestations are suggestive of a lower urinary tract disease but not specific, similar to the features of prostate hyperplasia. Elevated serum PSA is commonly seen in most patients. It may be caused by the rupture of some prostate glands in the enlarged cyst, leading to the release of PSA into the blood. Histologically, GMPC is typically composed of hyperplastic prostate glands and multiple cysts. The cysts are lined with stratified columnar and cuboidal cells. This case is consistent with previous reports.^[2,7,9]^ No atypical cell proliferation or mitosis was recognized. Immunohistochemical analysis showed the tumor cells were positive for PSA and PSAP staining, indicating a prostatic origin.

Preoperative imaging examination provides valuable tumor information. Due to the large size of GMPC, it is difficult to determine the origin of this retroperitoneal tumor by ultrasound. However, CT or MRI might reveal components of the tumor and its relationship with adjacent organs, which is crucial for the optimal determination of surgical procedure.^[8]^ GMPC is often characterized with multilocular masses in the pelvis. The septa in the cyst are often irregularly thickened. The tumor is mostly located between the rectum and bladder, whereas larger masses may extend beyond the pelvic cavity. The cysts display varied densities because of hemorrhage or mucin components. The relationship of tumor with adjacent organs may be revealed by contrast-enhanced CT scan. However, CT alone is not effective in making an accurate diagnosis. Due to its excellent soft tissue resolution, MRI may provide more information^[6,7,10]^ on the broader and compositions of the tumor. The surrounding tissue invaded or compressed by the tumor can be seen clearly in multiple MR sequences. Hemorrhage and mucin in cysts are easily seen in T1-weighted and T2-weighted images. Furthermore, the DWI reflects the cellularity and risk degree of the tumor. In our case, slightly higher SI on DWI and ADC maps suggested the benign nature of this tumor. Dynamic contrast-enhanced MRI (DCE-MRI) can also reflect the blood supply and boundary of tumors clearer than CT. Solid part of the tumor showed persistent enhancement on DCE-MRI, indicating the state of fibrous tissue of the tumor. Local invasion and lymph node involvement were not observed.

Differential diagnosis includes various cystic lesions arising from retroperitoneal space and pelvic cavity, such as prostatic cyst, prostatic abscess, prostate sarcoma, prostate cystadenocarcinoma, müllerian duct cysts, and cystic teratoma, and lymphangioma. Prostatic cyst and müllerian duct cysts are commonly asymptomatic and are often occasionally found during routine physical examination.^[16]^ They are often smaller than GMPC, with a diameter of less than 10 cm. They are easily recognized by their unilocular shape and nonenhancement of the cyst wall. The prostatic abscess is caused by prostate infection, the clinical symptoms of such cysts are always noticeable. On DWI, the SI indicates diffusion restriction in the abscess, which is different from appearance of GMPC.^[17]^ MRI can also be used to discriminate malignancies such as sarcoma and cystadenocarcinoma. These malignancies may present multiple cystic mass but more solid component than GMPC, markedly high SI on DWI and low SI on ADC are important for differential diagnosis.^[18]^ When fat, teeth, bones, or hair are present in a mass, the diagnosis of teratoma is easy because there may be fat, teeth, bones, or hair components in the mass, which is easily identified by imaging.

Although the final diagnosis of GMPC is made by histopathologic examination, imaging modalities especially MRI may also provide valuable information. In case of a giant multilocular prostatic mass with well-defined boundary and abundant vascularity, a benign feature on DWI, a differential diagnosis of GMPC should be considered.

## Author contributions

**Data curation:** Jingya Chen. Zhengjun Li, Jian Zhang.

**Investigation:** Hu Chen, Huiming WU. Yajing Wang.

**Methodology:** Wei Zhang, Zhongqiu Wang.

## Abbreviations

Abbreviations: ADC = apparent diffusion coefficient, CT = computed tomography, DCE-MRI = dynamic contrast-enhanced MRI, DWI = diffusion-weighted imaging, FPSA = free prostate-specific antigen, GMPC = giant multilocular prostatic cystadenoma, MRI = magnetic resonance imaging, PSA= prostate-specific antigen, SI = signal intensity, T1WI = T1-weighted imaging, T2WI = T2-weighted imaging, TPSA = total prostate-specific antigen.

## References

[1] Watanabe J Konishi T Takeuchi H. A case of giant prostatic cystadenoma. Hinyokika Kiyo 1990;36:1077–9. 2239615

[2] Nakamura Y Shida D Shibayama T. Giant multilocular prostatic cystadenoma. World J Surg Oncol 2019;17:42–51. 30808350 10.1186/s12957-019-1579-7 PMC6391754

[3] Park JP Cho NH Oh YT. Giant multilocular prostatic cystadenoma presenting with obstructive aspermia. Yonsei Med J 2007;48:554–6. 17594169 10.3349/ymj.2007.48.3.554 PMC2628090

[4] Ganesan S Ganesan K Joshi M. Giant multilocular prostatic cystadenoma. J Ultrasound Med 2006;25:795–8. 16731899 10.7863/jum.2006.25.6.795

[5] Choi YH Namkung S Ryu BY. Giant multilocular prostatic cystadenoma. J Urol 2000;163:246–7. 10604367

[6] Portugal Teixeira I Pereira PR Silva A. Giant multilocular prostatic cystadenoma, a diagnosis to consider in large pelvic male masses. Radiol Case Rep 2019;14:1473–7. 31641395 10.1016/j.radcr.2019.09.017 PMC6796614

[7] El-Asmar JM Saade C Dergham MYR. Multimodality approach to imaging giant multilocular cystadenoma of the prostate: a rare entity. Radiol Case Rep 2019;14:1117–22. 31338138 10.1016/j.radcr.2019.06.007 PMC6629975

[8] Abed El Rahman D Zago T Verduci G. Transperitoneal laparoscopic treatment for recurrence of a giant multilocular prostatic cystadenoma: a case report and review of the literature. Arch Ital Urol Androl 2016;88:66–7. 27072181 10.4081/aiua.2016.1.66

[9] Baad M Ericson K Yassan L. Giant multilocular cystadenoma of the prostate. Radiographics 2015;35:1051–5. 26172350 10.1148/rg.2015140316

[10] Olgun DC Onal B Mihmanli I. Giant multilocular cystadenoma of the prostate: a rare cause of huge cystic pelvic mass. Korean J Urol 2012;53:209–13. 22468219 10.4111/kju.2012.53.3.209 PMC3312072

[11] Chowdhury MM Abdulkarim JA. Case report. Multilocular cystadenoma of the prostate presenting as a giant pelvic mass. Br J Radiol 2009;82:e200–1. 19759205 10.1259/bjr/81852740

[12] Hauck EW Battmann A Schmelz HU. Giant multilocular cystadenoma of the prostate: a rare differential diagnosis of benign prostatic hyperplasia. Urol Int 2004;73:365–9. 15604586 10.1159/000081602

[13] Datta MW Hosenpud J Osipov V. Giant multilocular cystadenoma of the prostate responsive to GnRH antagonists. Urology 2003;61:225xxi–1225xxi. 12559312 10.1016/s0090-4295(02)02109-x

[14] Rusch D Moinzadeh A Hamawy K. Giant multilocular cystadenoma of the prostate. AJR Am J Roentgenol 2002;179:1477–9. 12438038 10.2214/ajr.179.6.1791477

[15] Kirsch AJ Newhouse J Hibshoosh H. Giant multilocular cystadenoma of the prostate. Urology 1996;48:303–5. 8753747 10.1016/S0090-4295(96)00178-1

[16] Quentin M Barski D Winter C. 3 T MRI of the prostate in patients with symptomatic prostatic utricle cyst. Urologe A 2010;49:1532–4. 21076809 10.1007/s00120-010-2451-0

[17] Starobinets O Kurhanewicz J Noworolski SM. Improved multiparametric MRI discrimination between low-risk prostate cancer and benign tissues in a small cohort of 5-alpha-reductase inhibitor treated individuals as compared with an untreated cohort. NMR Biomed 2017;30:5–15. 10.1002/nbm.3696 PMC5522750 28164396

[18] Dinis Fernandes C van Houdt PJ Heijmink S. Quantitative 3T multiparametric MRI of benign and malignant prostatic tissue in patients with and without local recurrent prostate cancer after external-beam radiation therapy. J Magn Reson Imaging 2019;50:269–78. 30585368 10.1002/jmri.26581 PMC6618021

